# Academic collaboration for health

**Published:** 2016

**Authors:** VL Purcarea

**Affiliations:** * "Carol Davila" University of Medicine and Pharmacy, Bucharest, Romania

The famous Professor Eliot Sorel from GWU, DHC and “Carol Davila” University of Medicine and Pharmacy in Bucharest affirmed that: “Health, healthcare and the transformations in the health systems need complementary innovations at the level of the professions in the health field, as far as education and training is concerned. Moreover, a new approach regarding health, healthcare, health and research systems is needed together with the creation of a new partnership between the bidders, consumers, decision makers, private, public and non-governmental sectors in all the countries, the economies with low, medium and high incomes”. 

In the welcoming note of the 4th Edition of “Carol Davila” University of Medicine and Pharmacy Congress, which took place in June at the Palace of Parliament, the distinguished Academician, Ioanel Sinescu, Rector of “Carol Davila” University of Medicine and Pharmacy in Bucharest, Romania, stated: “In a context of new beginnings, in order for this project to be continued, so as to keep the tradition of organizing this event alive, we need bold people, solidarity and common effort, which should be in agreement with a strategy that we should all build together. I am talking about the involvement of all the members of “Carol Davila” University of Medicine and Pharmacy in Bucharest, Romania”.

In order to materialize these ideas, “Carol Davila” University of Medicine and Pharmacy increased its valuable academic teaching staff with six remarkable personalities who are worldwide famous. 

**Fig. 1:**
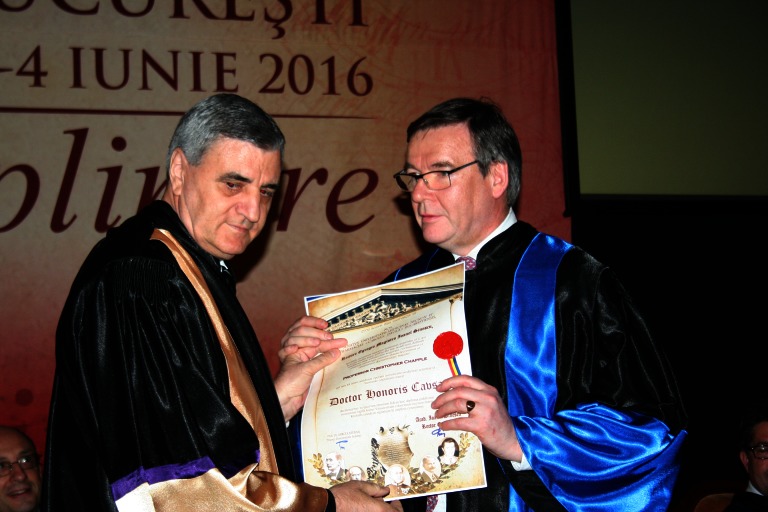
Prof. C. Chapple and Acad. Ioanel Sinescu

**Prof. C. Chapple**

Member of the Royal College of Surgeons in Great Britain (urological surgery field) and secretary general of the European Association of Urology, Professor Christopher Chapple graduated from the University of London as Bachelor of Medicine and Bachelor of Surgery. From the beginning, he had a keen interest in LUTS, female incontinence and reconstructive surgery, and has performed surgeries regarding the functional reconstruction of the urinary tract, later developing the European School of Urology on behalf of the European Association of Urology. 

In 2006, he was appointed Visiting Professor of Urology at the Sheffield Hallam University and in 2013, he became Honorary Professor of Urology at the University of Sheffield. 

Aside from being a brilliant surgeon, he is already responsible for more than 350 scientific articles published in prestigious journals and more than 50 books or chapters in the field of urology, with a great impact on the professional life and activity of the urological community. 

Moreover, he has also been acting as Editor in Chief of the journal “Neurourology and Urodynamics”, which is the official journal of the International Continence Society and the Section of Urodynamic and Female Urology of the American Urological Association, and, in 2011, he was awarded the St. Peter’s Medal of the British Association of Urological Surgeons. 

Professor Chapple was the first to localize the alpha-receptor subtypes within the human prostate, has thoroughly investigated the mechanisms involved in the alpha adrenergic and muscarinic innervations, urothelial relaxing factor, neurokinins and purinoceptors. He has ongoing projects on tissue engineering for autologous buccal mucosa for urethroplasty, and has been envisioning new approaches in the management of pelvic floor prolapse and stress urinary incontinence.

**Fig. 2:**
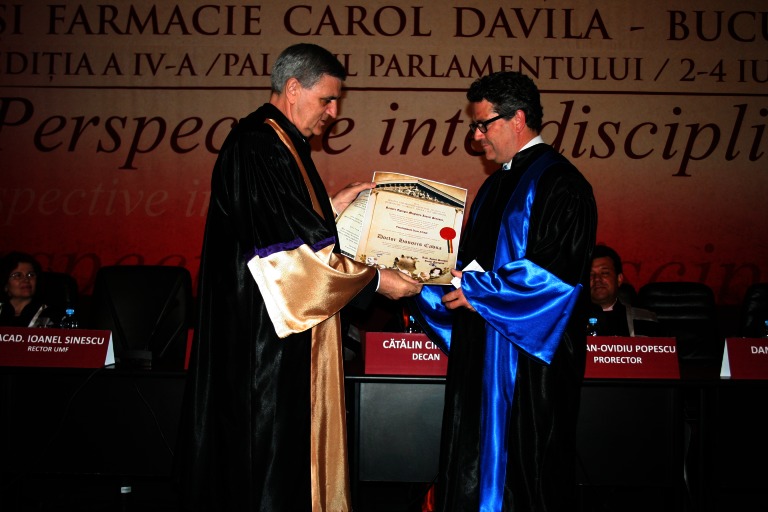
Prof. D. Atar and Acad. Ioanel Sinescu

**Prof. D. Atar**

Prof. D. Atar is the Head of Research at Oslo University Hospital, Norway. He holds a full Professorship in Cardiology at the University of Oslo, along with a Visiting Associate Professorship at the Johns Hopkins University, Baltimore, United States. He first went to Denmark, where he trained in internal medicine and cardiology at the University of Copenhagen. During 1990 and 1994, Professor Dan Atar moved to Baltimore, where he continued his career at the University of Maryland and the Johns Hopkins University, in the field of cardiology and as a researcher in heart problems. 

Later, he went to the University of Zurich, where he was trained in interventional cardiology, and where he became a cardiology faculty member, under the guidance of Thomas Lüscher, Editor in Chief of the European Heart Journal. His research focuses on myocardial biomarkers, myocardial function in myocardial infarction, particularly regarding the aspect of prevention of reperfusion injury, heart failure, and cardiovascular pharmacology. 

He has written more than 300 articles and book chapters and has more than 35,000 citations and a Hirsch Index of 56. At the same time, he is Associate Editor of journal “Cardiology” and member of the Editorial Board of the “European Heart Journal” and “Cardiovascular Diabetology”. 

Consequently to his outstanding activity, he received a number of Awards such as: Research Award of the International Society of Heart Research in 1994, Cardiology Award of the Swiss Heart Foundation in 1996, “Andreas Grüntzig Award in Interventional Cardiology” in 1997), and the Scientific Award of the Norwegian Society of Cardiology in 2014.

**Fig. 3:**
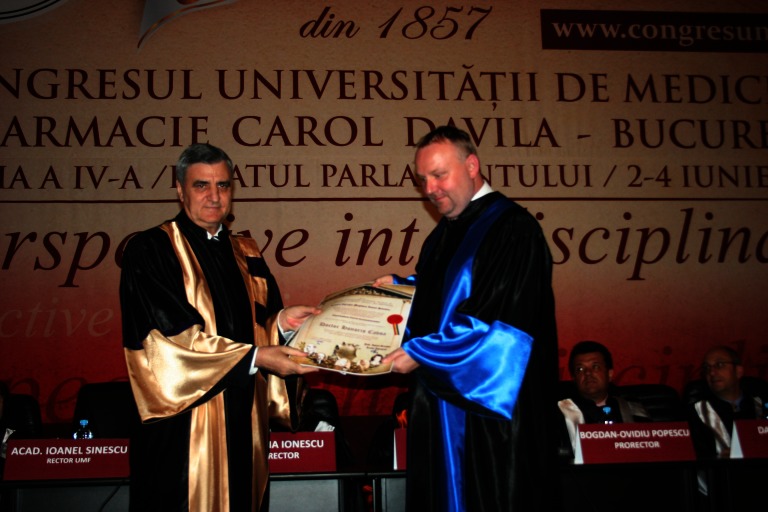
Prof. Piotr Radziszewski, MD, PhD and Acad. Ioanel Sinescu

**Prof. Piotr Radziszewski, MD, PhD**

Professor Piotr Radziszewski graduated from the School of Medicine – University of Medicine of Silesia, in Katowice, Poland.

He was Head of the Neurourology Research Unit in the Department of Urology of the Medical University of Warsaw, fellow of the European Board of Urology, Head of the Urogynecology and Functional Urology Unit and Chairman of the Department of General, Oncological, and Functional Urology of Medical University of Warsaw. 

The European Association of Urology acknowledged his interest and activity in the field of Neurourology and Urodynamics by appointing him as a member of the “Board of the Functional and Female Urology Section” of the European Association of Urology and as member of the “Guidelines Committee on Neurogenic Bladder”.

He is also a member of the Neurology Committee and of the Educational Committee of the International Continence Society. Professor Piotr Radziszewski published over 250 original articles in prestigious medical journals and authored 18 book chapters and 3 books. 

He is a member of the Editorial Board of many prestigious international medical publications such as “Neurourology and Urodynamics”, the “Central European Journal of Urology”, “Annals of Urology”, “Archives of Medical Sciences”, “Urologia Internationalis”.

In October 2015, the President of Poland, Andrew Duda, appointed Professor Piotr Radziszewski as a member of the Healthcare Committee of the National Development Council.

**Fig. 4:**
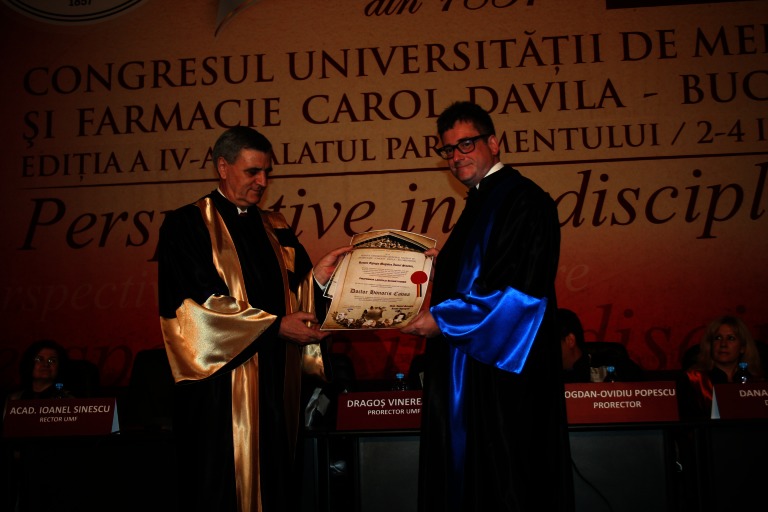
Prof. L. Schmetterer and Acad. Ioanel Sinescu

**Prof. L. Schmetterer**

Prof. L. Schmetterer graduated from the Technical University of Vienna in 1986 and University of Vienna Medical School in 1989. He is currently an Extraordinary Professor at the Centre of Medical Physics and Biomedical Engineering and at the Department of Clinical Pharmacology, Medical University of Vienna.

Professor Leopold Schmetterer has published over 270 original papers and reviews in Diabetes, Diabetes Care, Neurology, Ophthalmology, Progress in Retinal and Eye, his impact factor being of 10 and his works being cited over 11000 times. 

He is the author of a milestone book about Ocular Blood Flow (Springer, 2012) and coauthor of more than 30 book chapters.

He owns 16 patents, among them 10 in the field of OCT (Ocular Coherence Tomography).

He was President of EVER (European Association for Vision and Eye Research), member of the board of the Austrian Science Foundation and he is member of the editorial board of many international scientific journals including the highest ranked ophthalmological ones.

Recently, as recognition of his scientific contribution, it has been proposed to join one of the most famous and respected research institutions of the world: SERI (Singapore Eye Research Institute).

**Fig. 5:**
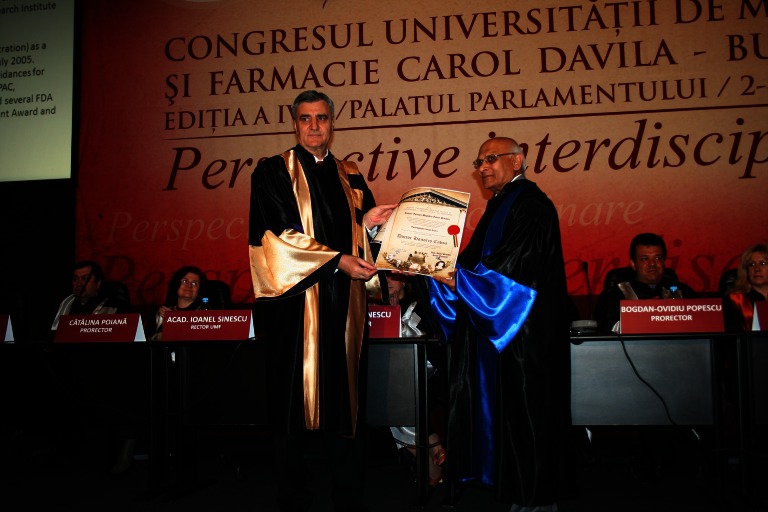
Prof. V. Shah, MD, PhD and Acad. Ioanel Sinescu

**Prof. V. Shah, MD, PhD**

Prof. Vinod Shah, MD, PhD is a Pharmacy graduate with Gold Medal from Madras University, India, worked for 5 years in the pharmaceutical industry, Sarabhai Chemicals, Baroda, India, and from 1975 joined US FDA (Food and Drug Administration) in the Division of Biopharmaceutics. He has received several FDA Awards including Award of Merit (1981, 1988), Scientific Achievement Award (2000), Commendable Service Award (2000) and Distinguished Career Service Award (2005). 

He is author/co-author of over 300 scientific papers and is a co-editor of five books. He was an Adjunct Professor at College of Pharmacy, University of Kentucky (2001-2011), a visiting professor at University of Philippines Manila, etc. Vinod Shah, MD, PhD is also an Honorary Member of Indian Pharmaceutical Association (2003), Honorary Member of Hungarian Society for Experimental and Clinical Pharmacology (2012). 

He is a recipient of FIP Industrial Pharmacy Award (2001 and 2008); IDMA - Eminent Pharmaceutical Analyst Award, India (2009) and SPDS Excellence Award, India (2014). 

He was the President of the American Association of Pharmaceutical Scientist (AAPS) in 2003 and a member of the International Affairs Committee of AAPS (2003-2014). 

**Fig. 6:**
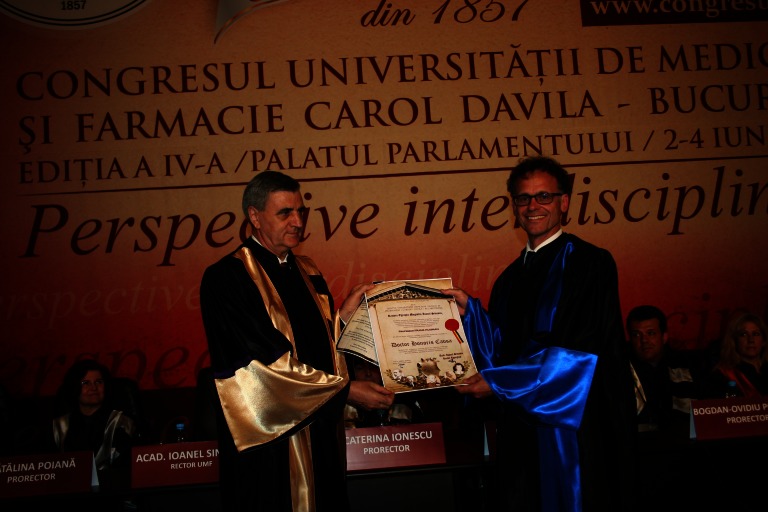
Prof. F. Huisman and Acad. Ioanel Sinescu

**Prof. F. Huisman**

Professor Frank Huisman is a member of Utrecht University in Netherlands. He was President of the European Association of History of Medicine (EAHMH). 

He started his academic career in the field of history of medicine and medical ethics at the University of Amsterdam, where he completed his PhD studies with a paper regarding the beginnings of the medical system and of modernity in Netherlands, which received the Lindeboom academic award. He continued his career in the Department of History of Medicine at the University of Maastricht and, in 2006, he became a Professor of History of Medicine at the University of Utrecht, where he currently activates. 

Professor Huisman is involved in the activity of one of the most important centers of scientific research in Europe, Descartes Centre for the History and Philosophy of the Sciences and the Humanities. 

The range of his interdisciplinary preoccupations in the field of history of medicine and of interdisciplinarity between medicine, society and human sciences, is impressive: history and ethics of health systems, analysis of the pharmaceutical infrastructure in medicine - De Nederlandse farmaceutische industrie in de negentiende en twintigste eeuw (with Rein Vos, Maastricht University), history of medical culture and of the concepts of medical school, theory and terminology of medicine. 

He has also made wide researches in the following fields: History, Philosophy, and Ethics of the sciences of life. 

Another excellence field is his preoccupation for opening histories and medical biographies in Europe in the context of scientific internationalization, which he highlighted with important, highly cited studies, such as Locating Medical History, The Stories and Their Meanings (with John Harley Warner, Yale University) and Dictionary of Medical Biography (the Netherlands and Flanders).

He is also actively involved in interdisciplinary research commissions in the University of Utrecht and the University of Maastricht - Historical and Comparative Studies of the Sciences and Humanities.

Moreover, Professor Huisman has many leading positions at the European and national level, being the Head of some prestigious medical organizations and societies. In addition, between 2009 and 2011 he was the President of the European Association of History of Medicine (EAHMH). 

He was also the unique author and co-author of many important books. The scientific studies published in the last 10 years alone prove the polyvalent mind of a true humanist scientist, recognized at the international level as an authority in the field.

**Executive Editor** **Prof. Dr. Eng. Victor Lorin Purcarea**

